# Patient and physician treatment preferences in relapsed/refractory follicular lymphoma: a discrete choice experiment in the United States, United Kingdom, France, Germany, Brazil, and Japan

**DOI:** 10.3389/fonc.2025.1589722

**Published:** 2025-07-10

**Authors:** John G. Gribben, Emmanuel Bachy, Markqayne Ray, Kathryn Krupsky, Kathleen Beusterien, Lewis Kopenhafer, Sara Beygi, Timothy Best, Graeme Ball, Oliver Will, Madhu Palivela, Anik Patel, Paola Ghione

**Affiliations:** ^1^ Barts Cancer Institute, Queen Mary University of London, London, United Kingdom; ^2^ Haematology Department, Hospices Civils de Lyon, Lyon, France; ^3^ Kite, A Gilead Company, Santa Monica, CA, United States; ^4^ Real World Evidence, Oracle Life Sciences, Austin, TX, United States; ^5^ Department of Medicine, Memorial Sloan Kettering Cancer Center, New York, NY, United States

**Keywords:** treatment preferences, relapsed/refractory, follicular lymphoma, discrete choice experiment, progression-free survival

## Abstract

**Introduction:**

The objectives of this study were to identify key treatment attributes that drive physician and patient preferences for second line (2L) and third line (3L) treatments in relapsed/refractory (R/R) follicular lymphoma (FL).

**Methods:**

A multi- country, internet-based survey was administered to patients(N=195) with R/R FL and treating physicians (N=300) from the United States, United Kingdom, France, Germany, Brazil, and Japan. The survey included two discrete choice experiments – one for 2L and one for 3L treatment options – that prompted respondents to select their preferred option between two hypothetical treatment profiles varying on seven attributes associated with treatment for R/RFL: progression-free survival (PFS), overall survival (OS), serious adverse events (AE), cytokine release syndrome (CRS) events, neurological events, fatigue, and administration. Mean preference weights and relative attribute importance were estimated in each sample, overall and by country, using hierarchical Bayesian models. Physician estimates were also stratified by practice setting.

**Results:**

Treatment preferences for physicians and patients were most influenced by PFS. Beyond PFS, patients placed greater emphasis on the administration of medications, whereas physicians tended to focus more on five-year OS and toxicity profiles of agents. Preference for PFS above all other 2L and 3L treatment attributes was consistent for physicians, regardless of practice setting and country. However, patient treatment preferences varied by country.

**Discussion:**

These results offer key perspectives on how physicians and patients evaluate treatment options in 2L and 3L treatment settings; this information is essential for facilitating shared decision-making in an expanding, complex treatment landscape.

## 1 Introduction

Follicular lymphoma (FL) is the most common indolent non-Hodgkin lymphoma globally (1). In the United States (US), incident FL cases are projected to rise from approximately 11,800 in 2020 to 14,700 in 2030, with similar increases expected in other regions (1). FL burdens patients, with notable impairments in health-related quality of life (HRQoL), working, and daily living, especially during later lines of therapy (2–9).

Although the outcome of first-line (1L) treatment of FL patients has improved in the era of chemo-immunotherapy and immunotherapy maintenance (10, 11), many patients experience refractory disease or have a disease recurrence within five years of receiving 1L therapy. Once patients develop relapsed or refractory (R/R) disease, the success of therapies decreases with each subsequent treatment line (1, 12–14), and in the second-line (2L) setting, roughly 70-75% of patients will have a subsequent relapse within five years (1, 15).

Clinical trial data evaluating the efficacy and safety of novel therapies, including CD-19 direct chimeric antigen receptor T-cell (CAR T) therapies, CD3 X CD20 bispecific antibodies, and EZH2, PI3K, and BTK inhibitors, demonstrate increases in the complete treatment response rate, even among pre-treated individuals (16). While an expanding treatment landscape for R/R FL is generally positive, increases in available treatments can also create challenges. Indeed, treatment selection for R/R FL is a nuanced process that increases in complexity as more therapeutic options become available and requires careful consideration of various factors (i.e., treatment goals, patient treatment history, and performance status (17). Perceptions of these factors can differ between patients and physicians (18, 19). Therefore, understanding how patients and physicians perceive treatment options is essential for shared decision-making.

The past two decades have demonstrated a growing focus on patient autonomy and the involvement of patients in treatment planning and decision-making. This complex and important process requires an understanding of patient preferences, both in terms of disease management and their overall health and includes addressing psychosocial needs in the context of cancer treatment (20). Evidence suggests that patients are more likely to adhere to medications and experience more positive outcomes when physicians engage their patients in treatment selection and share decision-making (21–27). Research evaluating patient and physician preferences for R/R FL treatment may produce knowledge that facilitates shared decision-making amidst an evolving and complex treatment landscape. However, robust preference research on R/R FL treatment is limited (28, 29), and extending such research to study designs that focus on later lines of treatment across diverse geographical regions may advance our understanding of patients’ treatment needs and promote shared decision-making in treatment selection.

The objectives of this study were to identify key treatment attributes that drive physician and patient preferences for 2L and third-line (3L) treatments in R/R FL and describe the congruence between patient and physician preferences.

## 2 Materials and methods

### 2.1 Study design

A cross-sectional, multi-country, online survey was administered to physicians between May and August 2023 and administered to patients with FL between September 2023 and January 2024. Participating countries included the US, the United Kingdom (UK), France, Germany Brazil, and Japan. The survey included two discrete choice experiments (DCE) to evaluate preferences for attributes associated with 2L and 3L treatment for R/R FL. The study protocol (10589-AMartin01) received exemption status from the Sterling Institutional Review Board on December 8, 2022. This study was conducted in accordance with the Declaration of Helsinki. All participants provided electronic consent and received fair-market compensation for their participation.

### 2.2 Participants

Recruitment was led by Global Perspectives, which specializes in online healthcare and health outcomes research. Physicians were recruited from an existing panel of healthcare providers who agreed to participate in online surveys. Patients were recruited through multiple sources, including patient databases, patient panels, social media, patient associations, and physician referrals. Recruitment quotas were applied to ensure a minimum number of physicians from academic and community settings in each country and a minimum number of patients from each country were included in our samples.

Physicians currently treating patients with FL, who treated patients with cancer for 3–40 years, and who treated at least five patients with FL in the past six months were eligible to participate in the study. Patients were required to be ≥18 years old, diagnosed with FL, and self-report that their FL had relapsed or was refractory unless the patient was from Japan. Due to recruitment challenges, patients from Japan were required to have received at least first-line treatment.

### 2.3 Survey content

**Figure 1:**
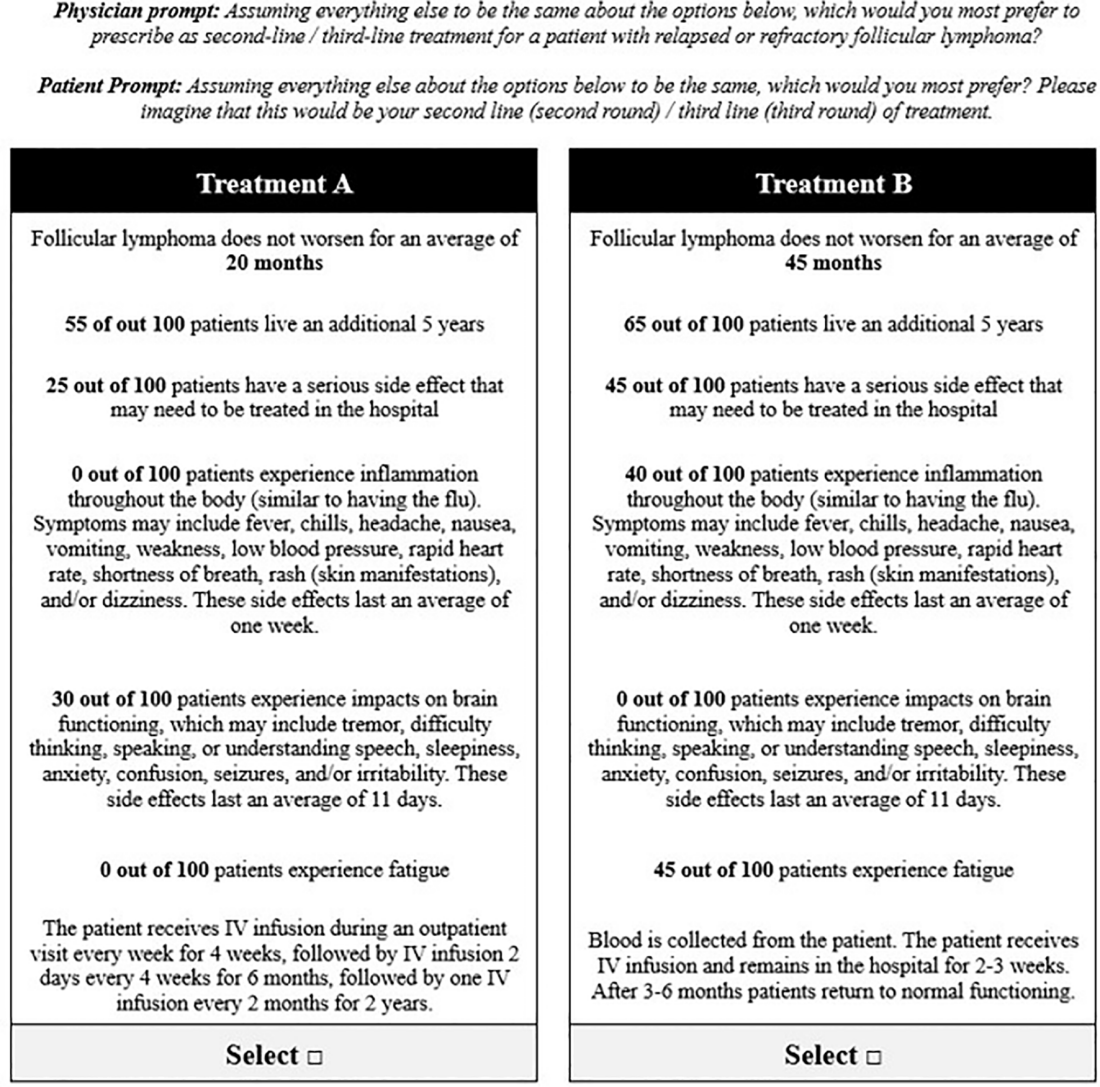
Example choice task from the discrete choice experiment to evaluate preferences for key treatment attributes.

Two DCE exercises (30–32) were conducted to evaluate preferences for 2L and 3L treatments in R/R FL, respectively. Each exercise included a series of 12 choice tasks, where respondents were instructed to select their preferred option from two hypothetical treatment profiles (**Figure 1**). For the 2L DCE, patients and physicians were asked to assume they were choosing (in the case of patients) or prescribing (in the case of physicians) 2L (second round)/2L treatment; in the 3L DCE, they were asked to assume they were choosing or prescribing 3L (third round)/3L treatment. The DCEs included attributes and levels associated with CAR Ts, bispecific antibodies, chemotherapy, and stem-cell transplantation. Specific therapies, like CAR T, are not currently available for R/R FL treatment in the UK and are not yet approved for 2L or 3L therapy in Europe. However, the hypothetical nature of the DCE allowed us to assess CAR T characteristics in these regions to provide insights into potential future treatment landscapes. Each hypothetical treatment profile consisted of seven attributes with three to six levels each: PFS, five-year overall survival (OS), serious adverse events (AE), cytokine release syndrome (any grade; CRS), neurological events (any grade), fatigue (any grade), and administration (**Table 1**). Attributes were identified via a focused literature review and refined with input from clinical experts. The experimental design for the DCEs was a balanced design with minimal overlap (31). The design was generated to optimize overall design efficiency in terms of (a) level balance (each level is shown approximately an equal number of times); (b) minimal level overlap (levels repeat within the same task); and (c) orthogonality (levels may be evaluated independently of other levels).

**Table 1:** Attributes and levels included in the second and third-line discrete choice experiments (DCE).

Label	Description for DCEs	Levels	
		Second Line DCE	Third Line DCE
Progression Free Survival (PFS)	Follicular lymphoma does not worsen for an average of _	1 year 8 months	10 months
		2 years 2 months	2 years
		3 years 9 months	3 years 3 months
Overall Survival (OS) at 5 years	_ patients live an additional 5 years	55 out of 100	43 out of 100
		65 out of 100	65 out of 100
		70 out of 100	74 out of 100
Serious Adverse Event (AE)	_ patients have a serious side effect that may need to be treated in the hospital	25 out of 100	27 out of 100
		35 out of 100	45 out of 100
		45 out of 100	58 out of 100
Cytokine Release Syndrome (CRS)	_ patients experience inflammation throughout the body (similar to having the flu). Symptoms may include fever, chills, headache, nausea, vomiting, weakness, low blood pressure, rapid heart rate, shortness of breath, rash (skin manifestations), and/or dizziness. These side effects last an average of one week.	0 out of 100	0 out of 100
		40 out of 100	45 out of 100
		78 out of 100	78 out of 100
Neurological AE of any grade	_ patients experience impacts on brain functioning, which may include tremor, difficulty thinking, speaking, or understanding speech, sleepiness, anxiety, confusion, seizures, and/or irritability. These side effects last an average of 11 days.	0 out of 100	0 out of 100
		30 out of 100	30 out of 100
		56 out of 100	56 out of 100
Fatigue any grade	_ patients experience fatigue	0 out of 100	0 out of 100
		25 out of 100	15 out of 100
		45 out of 100	30 out of 100
		–	45 out of 100
Administration	n/a	Blood is collected from the patient. 2–3 weeks later, the patient is admitted to the hospital to receive 3 days of IV infusion, followed by another IV infusion. Patients remain in hospital for an additional week. After 4 weeks patients return to normal functioning.	Blood is collected from the patient. 2–3 weeks later, the patient is admitted to the hospital to receive 3 days of IV infusion, followed by another IV infusion. Patients remain in hospital for an additional week. After 4 weeks patients return to normal functioning.
	n/a	Blood is collected from the patient. The patient receives IV infusion and remains in the hospital for 2–3 weeks. After 3–6 months patients return to normal functioning.	Blood is collected from the patient. The patient receives IV infusion and remains in the hospital for 2–3 weeks. After 3–6 months patients return to normal functioning.
	n/a	The patient receives IV infusion during an outpatient visit 2 days every 4 weeks for 6 months.	Blood is collected from the patient. The patient receives IV infusion and remains in the hospital for 2–4 weeks. After 6–12 months patients return to normal functioning. Patients are at risk for developing a serious condition that most often occurs within the first 100 days but can occur years after the procedure and often requires hospitalization.
	n/a	The patient receives IV infusion during an outpatient visit every week for 4 weeks, followed by IV infusion 2 days every 4 weeks for 6 months, followed by one IV infusion every 2 months for 2 years.	Tablet is taken by mouth twice a day for 2 years.
	n/a	–	The patient receives IV infusion during an outpatient visit every week for 3 weeks, followed by IV infusion every 3 weeks for one year.
	n/a	–	The patient receives IV infusion during an outpatient visit 2 days every 4 weeks for 6 months.

The treatment profiles shown in each DCE choice task were comprised of different combinations of attribute levels.

For the physician survey, sociodemographic characteristics, practice characteristics, and experiences treating patients with FL were collected. Patient sociodemographic, general health, clinical, and treatment characteristics were collected in the patient survey. The patient survey also included several patient-reported outcome measures (PROMs), including the Work Productivity and Activity Impairment (WPAI) – General Health Questionnaire (33), the EQ-5D utility index score, the EQ Visual Analogue Scale (VAS) (34), the Functional Assessment of Cancer Therapy – General (FACT-G) (35), and the Lymphoma Scale (36, 37) to supplement the FACT-G. A description of these instruments can be found in **Supplementary Methods**. Draft surveys were pilot tested via cognitive interviews (N=18 in each cohort) to ensure the survey content, including the DCEs, was clearly worded and performing as expected. The final physician and patient surveys took an average of 21.2 and 36.9 minutes to complete, respectively.

### 2.4 Statistical analysis

Descriptive statistics (mean and standard deviation [SD] for continuous variables; frequencies and percentages for categorical variables) were used to characterize the overall physician and patient samples. PROMs were also summarized descriptively.

To estimate preference weights for each attribute level in the DCEs, hierarchical Bayes (HB) multinomial logit models were utilized (30). These models generate utility estimates from individual-level choices and information from other respondents to estimate optimal coefficients. The prior distributions of the estimates are assumed to be normally distributed across the sample. HB models are considered hierarchical because they have two levels such that individual-level preference weights are described by a multivariate normal distribution at the higher level but governed by a multinomial logic model at the lower level. To ensure model estimates in the aggregate physician and patient samples were precise, *a priori* sample size calculations were completed using the formula (38), where *n* is the number of participants, *t* is the number of choice tasks, *a* is the number of profiles show in each task, and *c* represents the largest number of levels for any one attribute:


$$\frac{nta}{c}>500$$

With N=195 patients, seven attributes with a maximum of six levels, two alternatives per task, and 12 choice tasks, the formula results in 780, which is above 500, indicating that there was sufficient sample size to obtain relatively precise utility estimates for each of our DCE exercises in the patient cohort. With N=300 physicians and the same levels and tasks, the result is 1,200, again indicating that there was sufficient sample size for the physicians.

Mean preference weights (MPW) were estimated for each attribute level in the DCEs to measure relative preference. These weights measure relative preferences, such that the magnitude of the difference between attribute levels reflects how influential the difference has on treatment choice. MPWs allow for the assessment of trade-offs between utility gains and losses across attributes. As such, the difference between the highest (most preferred) and lowest (least preferred) MPW for each treatment indicates the magnitude of attributes influence on treatment preferences.

Relative importance (RI) for each attribute was also estimated using the MPWs to show the total impact on the utility of treatment associated with each attribute. RI estimates were derived at the respondent level by calculating the range of each attribute (utility of the most favorable minus the least favorable), then divided by the sum of all attribute ranges and multiplying by 100. Bivariate comparisons using one-way analysis of variance (ANOVA; α=0.05) were performed on RI estimates to examine unadjusted differences in physician treatment preferences, relative to academic setting (academic vs. community) and country. Given the smaller sample size for the patient cohort, descriptive comparisons of attribute RI were performed by country. All data were analyzed using Statistical Package for Social Sciences (SPSS) version 28 (IBM SPSS Statistics for Windows, Version 28.0, Armonk, NY: IBM Corp) for descriptive statistics and Sawtooth Software Lighthouse Studio version 9.12.0 or higher for the DCE analyses.

## 3 Results

### 3.1 Demographics

Participating physicians (N=300) were equally distributed across countries (**Table 2**). Mean (SD) age was 46.8 (9.6) years, with the oldest physicians in Japan (48.7 years) and the youngest in Brazil (39.2 years). More than half (54.3%) of physicians practiced in community settings, though this varied by country. Physicians had an average of 14.7 years of clinical experience treating FL (range: 10.5 years in Brazil to 20.3 years in Japan) and treated 44 patients, on average, with FL in the past six months.

**Table 2:** Physician characteristics.

	Overall (N=300)	US (n=50)	UK (n=50)	France (n=50)	Germany (n=50)	Brazil (n=50)	Japan (n=50)
Age in years; Mean (SD)	46.8 (9.6)	48.3 (12.3)	48.0 (7.8)	47.6 (7.5)	48.6 (8.0)	39.2 (7.8)	48.7 (9.9)
Practice Setting; n (%)							
Academic	137 (45.7%)	15 (30.0%)	39 (78.0%)	23 (46.0%)	27 (54.0%)	16 (32.0%)	17 (34.0%)
Community	163 (54.3%)	35 (70.0%)	11 (22.0%)	27 (54.0%)	23 (46.0%)	34 (68.0%)	33 (66.0%)
Years of clinical experience treating FL; Mean (SD)	14.7 (7.8)	14.7 (8.9)	12.9 (5.7)	15.0 (7.5)	14.8 (6.3)	10.5 (6.4)	20.3 (8.3)
Specialty^*^; n (%)							
Medical oncology	60 (20.0%)	17 (34.0%)	9 (18.0%)	10 (20.0%)	12 (24.0%)	8 (16.0%)	4 (8.0%)
Hematology oncology	189 (63.0%)	40 (80.0%)	39 (78.0%)	23 (46.0%)	43 (86.0%)	34 (68.0%)	10 (20.0%)
Hematology	107 (35.7%)	6 (12.0%)	13 (26.0%)	22 (44.0%)	8(16.0%)	19 (38.0%)	39 (78.0%)
Number of FL patients treated in the past 6 months (Mean; SD)	43.3 (55.1)	42.4 (37.2)	65.3 (88.5)	42.4 (56.6)	39.6 (39.5)	47.6 (56.8)	22.3 (17.9)
Proportion of patients with FL by age; Mean % (SD)							
20 years of age or younger	1.7 (4.1)	2.7 (5.4)	2.3 (4.7)	1.4 (3.6)	1.6 (3.6)	2.1 (4.3)	0.1 (0.7)
21–30 years of age	4.1 (6.5)	5.6 (7.1)	5.9 (7.8)	3.9 (5.7)	3.1 (4.3)	4.9 (8.2)	1.0 (3.5)
31–40 years of age	8.6 (8.2)	9.9 (8.4)	11.2 (9.2)	8.0 (6.1)	8.5 (7.3)	10.5 (9.8)	3.7 (5.2)
41–50 years of age	15.9 (10.5)	16.5 (10.7)	18.4 (12.8)	14.8 (7.6)	15.5 (7.3)	19.5 (11.8)	10.7 (9.3)
51–60 years of age	24.6 (11.4)	23.3 (7.9)	21.9 (10.3)	26.7 (10.3)	25.0 (10.3)	28.8 (15.6)	21.6 (11.1)
61–70 years of age	25.4 (12.5)	25.8 (12.9)	22.0 (11.2)	26.6 (13.3)	27.2 (13.3)	21.1 (12.0)	29.8 (10.3)
>70 years of age	19.8 (15.3)	16.3 (13.2)	18.3 (13.0)	18.6 (12.7)	19.2 (13.0)	13.1 (11.2)	33.1 (19.6)
Number of treated patients with FL by line of therapy within past 6 months; Mean (SD)							
1L	42.3 (16.5)	43.7 (16.5)	41.8 (15.9)	40.0 (13.5)	41.9 (16.3)	42.6 (14.3)	43.7 (21.5)
2L	26.6 (9.3)	26.8 (8.3)	26.1 (6.3)	26.5 (9.2)	27.3 (8.4)	26.1 (8.3)	26.6 (14.0)
3L	19.2 (18.1)	15.8 (8.7)	15.5 (7.5)	17.6 (8.5)	17.3 (7.9)	16.5 (7.8)	14.8 (8.4)
4L	7.6 (7.3)	8.2 (7.2)	8.3 (7.1)	9.5 (7.6)	7.7 (6.9)	6.8 (6.7)	5.3 (8.1)
Not treated yet	7.3 (13.1)	5.5 (11.4)	8.2 (14.8)	6.5 (9.0)	5.8 (8.7)	8.1 (13.4)	9.5 (18.7)

FL, follicular lymphoma; SD, standard deviation; US, United States; UK, United Kingdom.*Physicians could select more than one specialty. Therefore, counts may not sum to the total sample size and percentages may not sum to 100%.

Participating patients (N=195) included those from the US (n=50, 25.6%), Germany (n=40, 20.5%), the UK (n=35, 17.9%), Brazil (n=30, 15.4%), France (n=25, 12.8%), and Japan (n=15, 7.7%) (**Table 3**). Women comprised a large proportion of patients (59.0%; range: 54.3% in the UK to 65.0% in Germany) and the mean (SD) age of patients was 59.2 (10.2) years. The mean (SD) number of months since patients were diagnosed with FL was 80.6 (60.8). The time since receiving the first FL treatment was the shortest in the US (31.9 [18.4] months) and the longest in France (122.4 [101.4]). The average number of FL treatments that patients received was 2.6 (SD=1.3) and 39.5% of patients said their FL returned within 2 years after starting their first FL treatment.

**Table 3:** Patient clinical and demographic characteristics.

	Overall	US (n=50)	UK (n=35)	France (n=25)	Germany (n=40)	Brazil (n=30)	Japan (n=15)
	N=195						
Age in years; Mean (SD)	59.2 (10.2)	60.8 (4.6)	63.4 (9.1)	66.8 (7.3)	56.9 (9.6)	47.7 (12.5)	59.9 (6.7)
Gender; n (%)							
Male	80 (41.0%)	20 (40.0%)	16 (45.7%)	12 (48.0%)	14 (35.0%)	12 (40.0%)	6 (40.0%)
Female	115 (59.0%)	30 (60.0%)	19 (54.3%)	13 (52.0%)	26 (65.0%)	18 (60.0%)	9 (60.0%)
Marital Status; n (%)							
Committed relationship/Married	144 (73.9%)	38 (76.0%)	24 (68.6%)	20 (80.0%)	27 (67.5%)	25 (83.3%)	10 (66.7%)
Single, never married, separated/divorced, or widowed	37 (19.0%)	4 (8.0%)	10 (28.6%)	5 (20.0%)	8 (20.0%)	5 (16.7%)	5 (33.3%)
Declined to answer	14 (7.2%)	8 (16.0%)	1 (2.9%)	0 (0.0%)	5 (12.5%)	0 (0.0%)	0 (0.0%)
Location of Residence; n (%)							
Major metropolitan area	68 (34.9%)	4 (8.0%)	6 (17.1%)	0 (0.0%)	24 (60.0%)	28 (93.3%)	6 (40.0%)
Urban area	35 (17.9%)	17 (34.0%)	7 (20.0%)	3 (12.0%)	2 (5.0%)	2 (6.7%)	4 (26.7%)
Suburb of a large city	33 (16.9%)	17 (34.0%)	12 (34.3%)	1 (4.0%)	1 (2.5%)	0 (0.0%)	2 (13.3%)
Small city	31 (15.9%)	8 (16.0%)	4 (11.4%)	8 (32.0%)	8 (20.0%)	0 (0.0%)	3 (20.0%)
Rural or small town	28 (14.4%)	4 (8.0%)	6 (17.1%)	13 (52.0%)	5 (12.5%)	0 (0.0%)	0 (0.0%)
Months since FL was diagnosed; Mean (SD)	80.6 (60.8)	35.3 (18.4)	87.3 (55.2)	134.3 (100.6)	105.0 (50.4)	66.9 (21.3)	51.9 (31.3)
Top Comorbidities; n (%)							
High blood pressure (hypertension)	70 (35.9%)	15 (30.0%)	18 (51.4%)	1 (4.0%)	23 (57.5%)	12 (40.0%)	1 (6.7%)
Asthma	31 (15.9%)	14 (28.0%)	5 (14.3%)	0 (0.0%)	0 (0.0%)	8 (26.7%)	4 (26.7%)
Diabetes	20 (10.3%)	6 (12.0%)	1 (2.9%)	0 (0.0%)	6 (15.0%)	6 (20.0%)	1 (6.7%)
Osteoporosis or other bone disease	17 (8.7%)	2 (4.0%)	3 (8.6%)	0 (0.0%)	5 (12.5%)	7 (23.3%)	0 (0.0%)
Heart disease	10 (5.1%)	0 (0.0%)	8 (22.9%)	1 (4.0%)	0 (0.0%)	1 (3.3%)	0 (0.0%)
Healthcare professional managing FL; n (%)							
GP/FP/Internist	3 (1.5%)	0 (0.0%)	0 (0.0%)	2 (8.0%)	1 (2.5%)	0 (0.0%)	0 (0.0%)
Hematologist	71 (36.4%)	2 (4.0%)	32 (91.4%)	18 (72.0%)	4 (10.0%)	1 (3.3%)	14 (93.3%)
Oncologist/Medical Oncologist	116 (59.5%)	48 (96.0%)	1 (2.9%)	4 (16.0%)	34 (85.0%)	28 (93.3%)	1 (6.7%)
Radiation Oncologist	2 (1.0%)	0 (0.0%)	0 (0.0%)	0 (0.0%)	1 (2.5%)	1 (3.3%)	0 (0.0%)
Another type of healthcare professional	2 (1.0%)	0 (0.0%)	2 (5.7%)	0 (0.0%)	0 (0.0%)	0 (0.0%)	0 (0.0%)
Not Sure	1 (0.5%)	0 (0.0%)	0 (0.0%)	1 (4.0%)	0 (0.0%)	0 (0.0%)	0 (0.0%)
Months since receiving first FL treatment; Mean (SD)	70.0 (59.3)	31.9 (18.4)	73.6 (58.2)	122.4 (101.4)	86.5 (47.0)	61.4 (17.5)	36.3 (26.9)
Number of FL treatments received; Mean (SD)	2.6 (1.3)	3.0 (1.2)	2.6 (0.9)	3.1 (1.7)	2.6 (1.5)	1.9 (0.8)	1.6 (1.2)
Treatments ever taken to manage FL; n (%)							
Rituxan or rituximab as monotherapy or in combination	85 (43.6%)	29 (58.0%)	11 (31.4%)	5 (20.0%)	25 (62.5%)	13 (43.3%)	2 (13.3%)
Rituxan or rituximab in combination with another drug	134 (68.7%)	43 (86.0%)	25 (71.4%)	18 (72.0%)	23 (57.5%)	15 (50.0%)	10 (66.7%)
Immunotherapy and/or chemotherapy (not including rituximab)	72 (36.9%)	31 (62.0%)	9 (25.7%)	4 (16.0%)	17 (42.5%)	6 (20.0%)	5 (33.3%)
PI3K Inhibitors	15 (7.7%)	13 (26.0%)	1 (2.9%)	0 (0.0%)	0 (0.0%)	1 (3.3%)	0 (0.0%)
Radiation	35 (18.0%)	10 (20.0%)	4 (11.4%)	4 (16.0%)	16 (40.0%)	1 (3.3%)	0 (0.0%)
Chimeric Antigen Receptor T Cell Therapy (CAR T)	3 (1.5%)	0 (0.0%)	0 (0.0%)	3 (12.0%)	0 (0.0%)	0 (0.0%)	0 (0.0%)
Bone marrow/stem cell transplantation	8 (4.1%)	0 (0.0%)	4 (11.4%)	3 (12.0%)	1 (2.5%)	0 (0.0%)	0 (0.0%)
FL returned in 2 years (yes); n (%)	77 (39.5%)	29 (58.0%)	11 (31.4%)	9 (36.0%)	8 (20.0%)	19 (63.3%)	1 (6.7%)

FL, follicular lymphoma; GP, general practitioner; FP, family practitioner; SD, standard deviation; US, United States; UK, United Kingdom.

#### 3.1.1 Patient-reported outcome measures

Among all patients, the mean (SD) percentage of absenteeism, presenteeism, overall work productivity impairment, and overall activity impairment over the past seven days was 35.2 (36.2), 38.2 (24.7), 49.3 (29.6), and 42.5 (24.1), respectively (**Table 4**). Patients from Japan reported the lowest levels of work productivity and activity impairment, across all metrics. Patients from France, on average, reported the highest levels of presenteeism (53.3%) and overall work productivity impairment (62.4%). Patients from Germany experienced the most absenteeism (62.4%), whereas patients from Brazil experienced the highest percentage of overall activity impairment (53.7%).

**Table 4:** Patient-reported outcome measures for patients in the overall sample and by country.

	All Patients (N=195)	US (n=50)	UK (n =35)	France (n =25)	Germany (n =40)	Brazil (n =30)	Japan (n =15)
	Mean (SD)	Mean (SD)	Mean (SD)	Mean (SD)	Mean (SD)	Mean (SD)	Mean (SD)
Work Productivity and Activity Impairment							
Absenteeism^1^ (N=87)	35.2 (36.2)	52.4 (41.9)	19.6 (34.7)	17.2 (18.4)	62.4 (44.4)	35.5 (20.4)	0.0 (0.0)
Presenteeism^2^ (N=74)	38.2 (24.7)	33.8 (13.0)	28.8 (25.8)	53.3 (33.9)	42.2 (23.3)	48.2 (17.6)	12.5 (24.4)
Overall workplace productivity loss^3^ (N=74)	49.3 (29.6)	50.6 (25.3)	32.9 (31.3)	64.8 (19.8)	55.1 (30.9)	64.1 (17.6)	12.5 (24.4)
Activity impairment^4^	42.5 (24.1)	40.2 (21.7)	38.0 (20.7)	39.2 (31.5)	53.0 (17.7)	53.7 (18.1)	16.0 (26.1)
Health-Related Quality of Life							
EQ-5D Index^5^	0.706 (0.192)	0.732 (0.157)	0.750 (0.156)	0.679 (0.299)	0.706 (0.200)	0.574 (0.084)	0.826 (0.159)
EQ VAS^6^	63.8 (15.6)	64.0 (12.6)	63.7 (16.1)	73.6 (15.4)	52.8 (14.5)	64.6 (11.4)	74.3 (16.1)
FACT - G^7^	67.4 (17.9)	63.9 (21.1)	70.3 (16.9)	71.4 (14.6)	59.8 (16.3)	74.0 (16.1)	72.2 (13.5)
FACT - Lymphoma Total^8^	104.7 (27.5)	103.4 (32.2)	108.2 (24.9)	115.8 (22.4)	87.3 (25.4)	110.4 (22.7)	117.2 (16.6)

FACT, Functional Assessment of Cancer Therapy; SD, standard deviation; US, United States; UK, United Kingdom; VAS, Visual Acuity Score.

^1^Absenteeism was calculated among patients who participated in the workforce at the time of the survey and worked >0 hours and missed > 0 hours of work in the 7 days prior. Scores range from 0 to 100 where higher scores indicate greater work absenteeism.

^2^Presenteeism was calculated for patients who participated in the workforce at the time of the survey and who worked >0 hours in the last 7 days. Scores range from 0 to 100 where higher scores indicate greater work presenteeism (i.e., impaired work time).

^3^Overall workplace productivity loss was calculated for patients who had a non-missing absenteeism and presenteeism score. Scores range from 0 to 100 where higher scores indicate greater overall work productivity impairment.

^4^Activity impairment ranges from 0 to 100 where higher scores indicate more activity impairment.

^5^EQ-5D Index score is an indicator of quality of life that ranges from 0 to 1, with higher scores indicating better quality of life.

^6^EQ-VAS ranges from 0 to 100 where higher scores indicate better quality of life.

^7^FACT - G is a composite of physical, social, emotional, and functional scales and ranges from 0–108 where higher scores indicate better outcomes.

^8^FACT - Lymphoma total score is a composite of physical, social, emotional, functional, and lymphoma symptom scales and ranges from 0–168 where higher scores indicate better outcomes.

The mean (SD) EQ-5D index score ranged from 0.574 (0.084) in Brazil to 0.826 (0.159) in Japan, with an overall score of 0.706 (0.192) in the pooled patient sample (**Table 4**). The mean (SD) EQ VAS was 63.8 (15.6) in the pooled patient sample, with the lowest values noted among patients from Germany (52.8 [14.5]) and the highest from Japan (74.3 [16.1]). The mean (SD) FACT-G score was 67.4 (17.9) in the overall patient sample (range: 59.8 [16.3] in Germany to 72.2 [13.5] in Japan). When the Lymphoma Subscale was factored into HRQoL, the overall patient cohort had a mean (SD) score of 104.7 (27.5), with Germany patients once again having the lowest score on average (87.3 [25.4]) and Japan patients having the highest average score (117.2 [16.6]).

### 3.2 2L DCE findings

**Figure 2:**
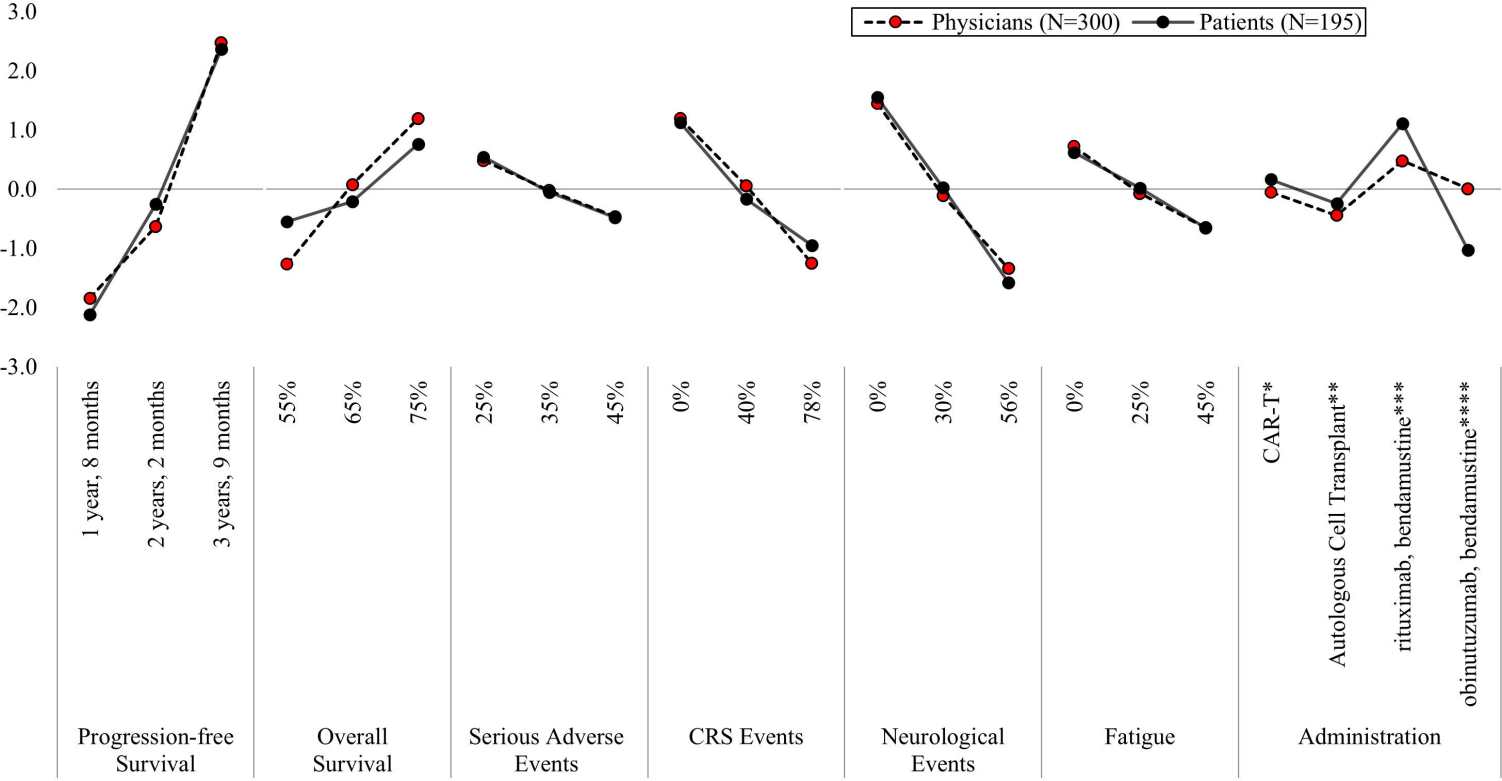
Mean preference weights for attributes associated with second-line treatment for relapsed/refractory follicular lymphoma. CRS, Cytokine Release Syndrome; IV, intravenous; PFS, progression free survival. Preference weights should not be interpreted by themselves. Instead, the magnitude of change within one attribute should be compared to change within another attribute. All preference weights of levels within an attribute sum to 0. *Blood is collected from the patient. 2–3 weeks later, the patient is admitted to the hospital to receive 3 days of IV infusion, followed by another IV infusion. Patients remain in the hospital for an additional week. After 4 weeks patients return to normal functioning. **Blood is collected from the patient. The patient receives IV infusion and remains in the hospital for 2–3 weeks. After 3–6 months patients return to normal functioning. ***The patient receives IV infusion during an outpatient visit 2 days every 4 weeks for 6 months. ****The patient receives IV infusion during an outpatient visit every week for 4 weeks, followed by IV infusion 2 days every 4 weeks for 6 months, followed by one IV infusion every 2 months for 2 years.

On average, both physician and patient treatment preferences were most influenced by increases in PFS, followed by decreases in the risk of neurological events, compared to other treatment attributes (**Figure 2**, **Supplementary Table 1**). Among physicians, increasing PFS from one year, eight months (MPW = -1.84) to three years, nine months (MPW = 2.48), reflects an absolute difference of 4.32, whereas decreasing risk of neurological events from 56% (MPW = -1.34) to 0% (MPW = 1.45) reflects an absolute difference of 2.79. Similarly, for patients, the absolute difference in increasing PFS from one year, eight months (MPW = -2.12) to three years, nine months (MPW = 2.37) was 4.49, and decreasing risk of neurological events from 56% (MPW = -1.58) to 0% (MPW = 1.55) was 3.13. As such, both physicians and patients were willing to accept an increased neurological event risk of 56% in exchange for an increase in PFS of 25 months.

Following PFS and risk of neurological events, physician treatment preferences were most influenced by increasing five-year OS from 55% (MPW = -1.27) to 75% (MPW = 1.19) and decreases in the CRS risks from 78% (MPW = -1.25) to 0% (MPW = 1.19) (**Figure 2**, **Supplementary Table 1**). In contrast, patients prioritized treatment administration over reductions in CRS risks and five-year OS improvements. Patients preferred treatments administered in an outpatient setting on two days, every four weeks for six months (MPW = 1.11) over an outpatient infusion with extended follow-up treatments every two months for up to two years (MPW = -1.03). For physicians, administration had the least influence on 2L treatment preferences overall, compared to the other attributes.

### 3.3 3L DCE findings

**Figure 3:**
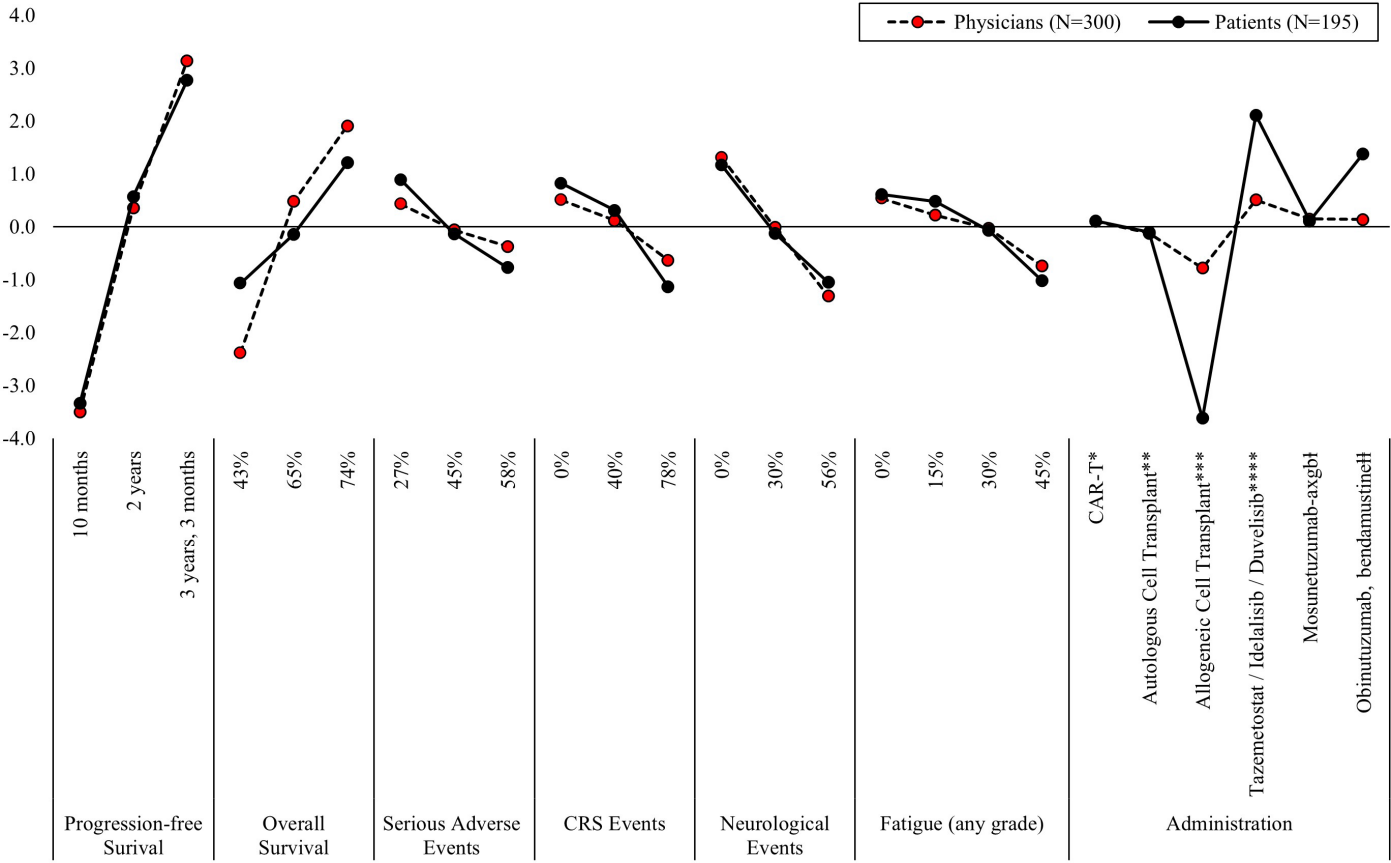
Mean preference weights for attributes associated with third-line treatment for relapsed/refractory follicular lymphoma. PFS, progression free survival; CRS, cytokine release syndrome. Note: Preference weights should not be interpreted by themselves. Instead, the magnitude of change within one attribute should be compared to change within another attribute. All preference weights of levels within an attribute sum to 0. *Blood is collected from the patient. 2-3 weeks later, the patient is admitted to the hospital to receive 3 days of IV infusion, followed by another IV infusion. Patients remain in the hospital for an additional week. After 4 weeks patients return to normal functioning. **Blood is collected from the patient. The patient receives IV infusion and remains in the hospital for 2-3 weeks. After 3-6 months patients return to normal functioning. ***Blood is collected from the patient. The patient receives IV infusion and remains in the hospital for 2-4 weeks. After 6-12 months patients return to normal functioning. Patients are at risk for developing a serious condition that most often occurs within the first 100 days but can occur years after the procedure and often requires hospitalization. ****Tablet is taken by mouth twice a day for 2 years. ⱡThe patient receives IV infusion during an outpatient visit every week for 3 weeks, followed by IV infusion every 3 weeks for one year. ⱡⱡThe patient receives IV infusion during an outpatient visit 2 days every 4 weeks for 6 months.

For both physicians and patients in the 3L setting, increasing PFS from 10 months (MPW for physicians = -3.50 and patients = -3.34) to three years and three months (MPW for physicians = 3.14 and patients = 2.77) most influenced treatment preferences (**Figure 3**, **Supplementary Table 2**). While increasing five-year OS from 43% (MPW = -2.38) to 74% (MPW = 1.91) was the second most influential attribute for physicians’ treatment preferences, treatment administration was the next most influential treatment attribute for patients. Patients least preferred the administration method of allogeneic stem cell transplant (ASCT), which carries a risk of developing acute graft versus host disease, a serious condition that typically occurs within the first 100 days and requires hospitalization (MPW = -3.62). Instead, patients preferred the option of taking oral medication twice a day for two years (MPW = 2.14). Patient 3L treatment preferences were most influenced by increasing five-year OS from 43% (MPW = -1.07) to 74% (MPW = 1.21) and reducing the risk of neurological events from 56% (MPW = -1.05) to 0% (MPW = 1.17).

Beyond PFS and OS, the risk of neurological events and type of administration were among the most influential treatment attributes for physicians (**Figure 3**, **Supplementary Table 2**). Like patients, physicians least preferred ASCT (MPW = -0.78) and most preferred oral tablets twice a day for two years (MPW = 0.51), though the method of administration was not as important to physicians relative to patients.

Due to the influence of the ASCT on patient 3L treatment preferences, we conducted a *post-hoc* sensitivity analysis where the ASCT attribute level was removed from the model (**Supplementary Table 3**, **Supplementary Figure 1**). After removal, administration was the least influential 3L treatment attribute on physician preference, relative to the others assessed. For patients, the overall influence of administration on 3L treatment preferences decreased, such that the influence of administration was roughly equal to that of five-year OS, and the risk of neurological events (evidenced by the absolute difference between the most and least preferred attribute levels).

### 3.4 Subgroup comparisons of physician treatment preferences

**Figure 4:**
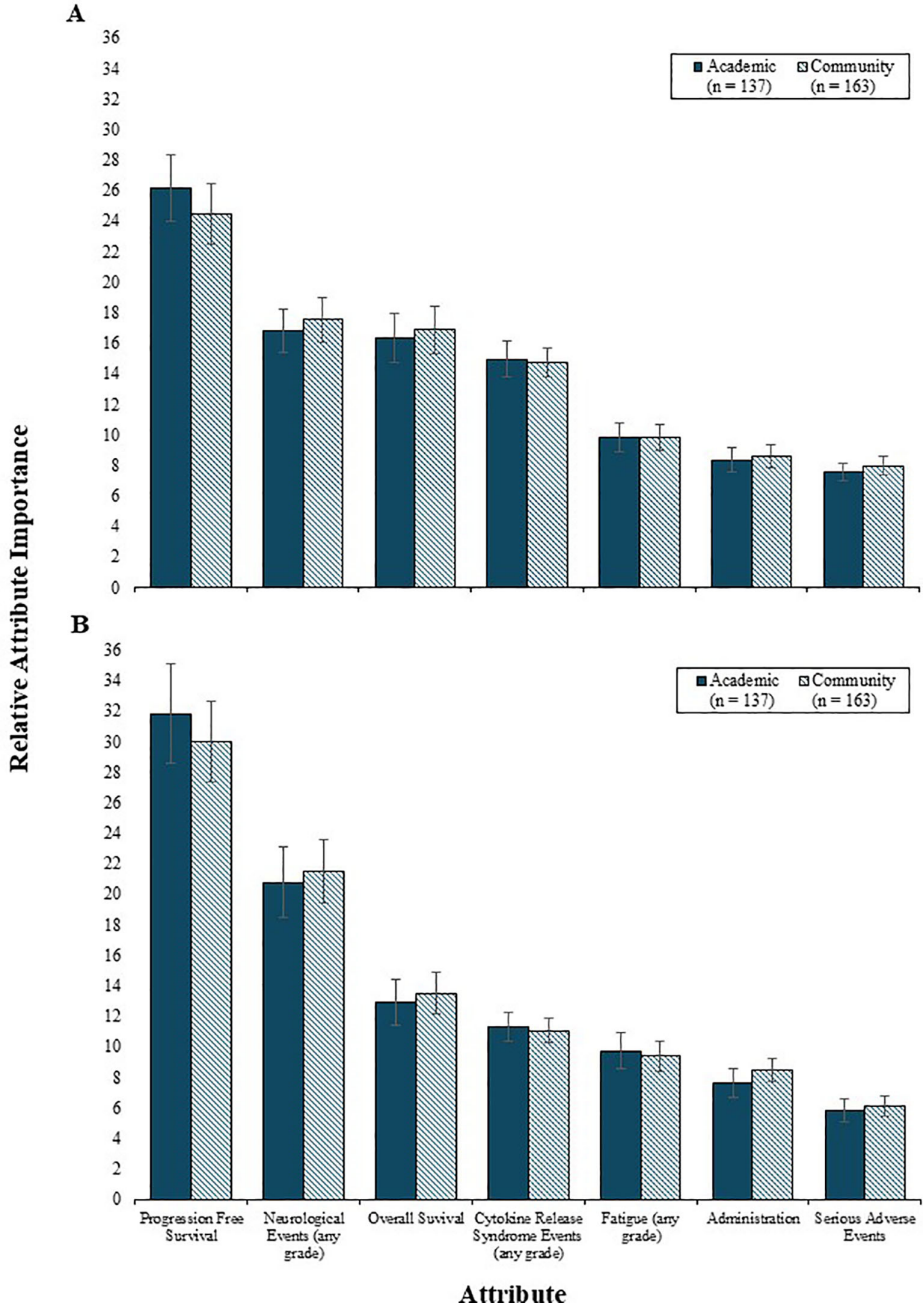
The relative importance of 2L **(A)** and 3L **(B)** treatment attributes by physician treatment setting. 2L, second line; 3L, third line.

**Figure 5:**
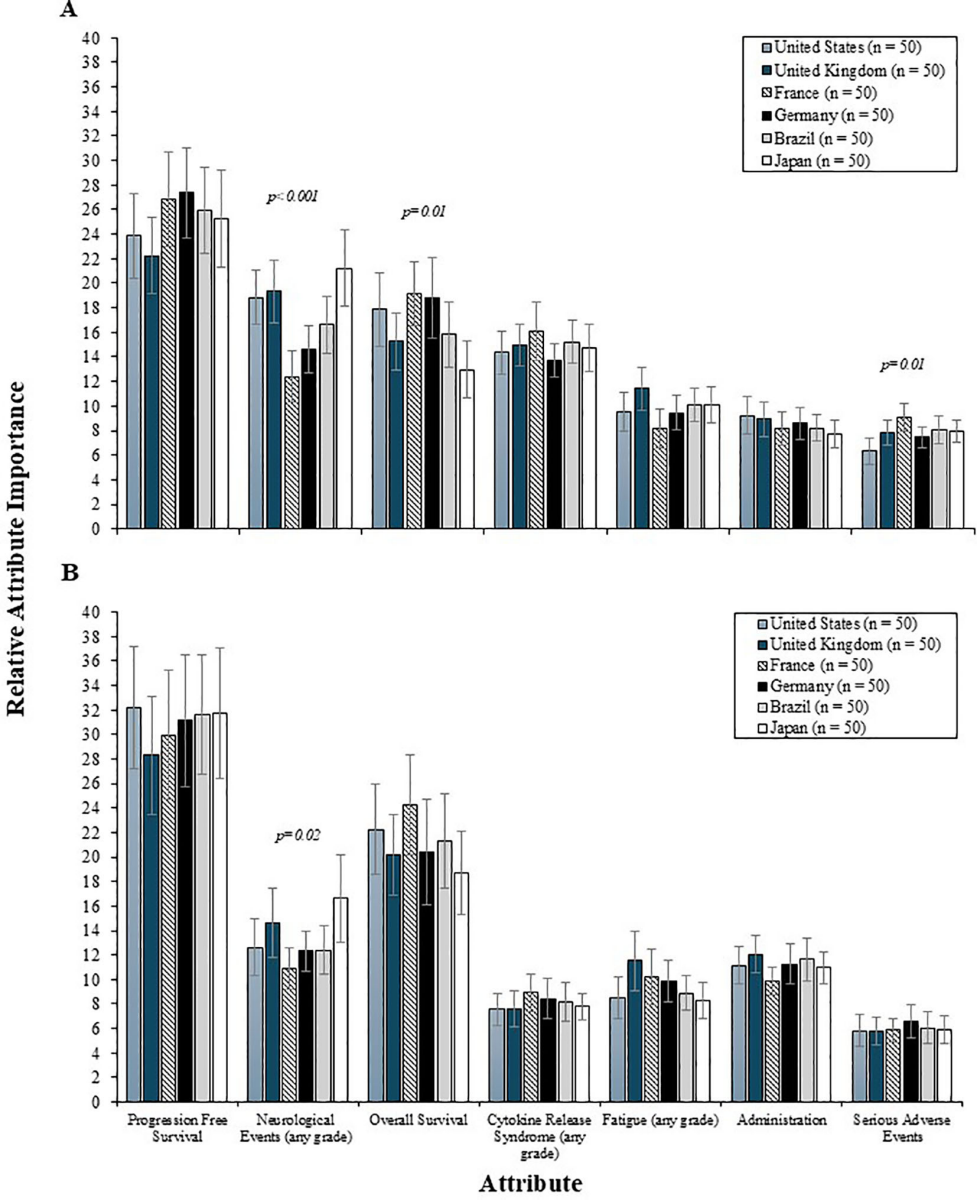
The relative importance of 2L **(A)** and 3L **(B)** treatment attributes among physicians by country. 2L, second line; 3L=third line.

Physician preferences for 2L and 3L treatment attributes were comparable between community and academic settings (i.e., all *p* values were >0.05) (**Figure 4**), with PFS being the most influential and equally valued by both groups. The influence of PFS was also consistent across countries, for both 2L and 3L settings (**Figure 5**). However, variation by country was noted in the 2L RI estimates for neurological events (p<0.001), OS (p<0.05), and serious AEs (p<0.05). Specifically, among physicians from France and Germany, the RI of neurological events was lower than other countries, such that both OS and CRS events were perceived as being relatively more important than neurological events. Consequently, the RI of OS in the 2L setting was highest among physicians in France and Germany. While serious AEs were consistently the least important 2L treatment attribute relative to others assessed, the magnitude of influence of serious AEs on treatment preferences was higher among physicians from France (RI = 9.1) and lowest among those from the US (RI = 6.3). In the 3L setting, the relative importance of the risk of neurological events varied by country (p<0.05), such that the relative importance was lowest among physicians from France (RI=10.9) and highest among those from the US (RI=16.6).

### 3.5 Subgroup comparisons of patient treatment preferences

**Figure 6:**
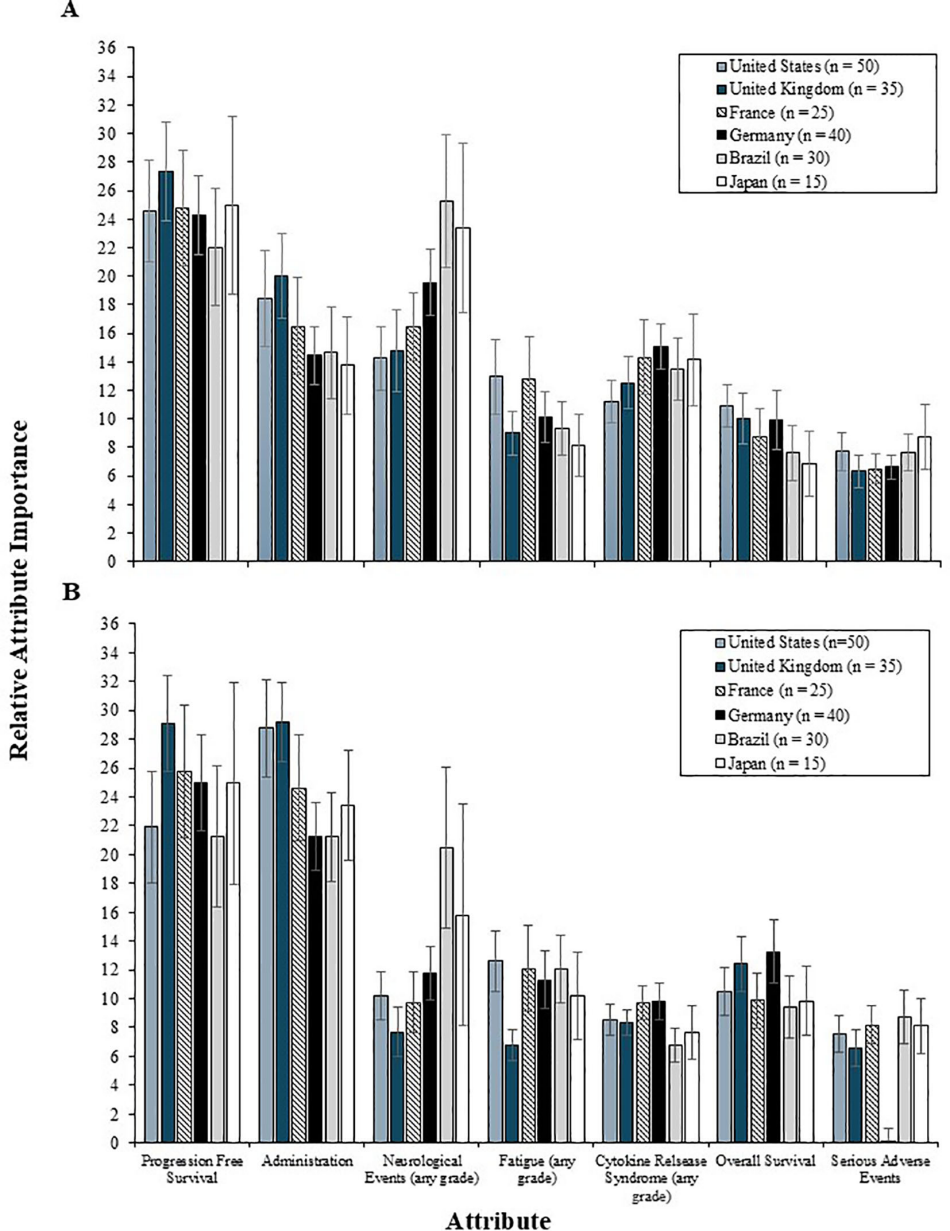
The relative importance of 2L **(A)** and 3L **(B)** treatment attributes among patients by country. 2L, second line; 3L, third line.

In the 2L setting, PFS was the most important attribute, relative to the others among patients from all countries except Brazil, who placed greater value on reducing the risk of neurological events (RI = 25.3) over increases in PFS (RI = 22.0) (**Figure 6**). For most countries, administration and reducing the risk of neurological events were among the most important 2L treatment attributes, whereas OS and serious AEs were consistently among the least important attributes.

In the 3L setting, the relative importance of PFS and administration was similar to that of patients (**Figure 6**). Patients from the US found administration relatively more important (RI = 28.8) than PFS (RI = 21.9), whereas patients from France, Germany, and Japan found PFS most important (RI = 25.8, 25.0, and 24.9, respectively), followed by administration (RI = 24.6, 21.3, and 23.4, respectively). Patients from the UK and Brazil weighted PFS and administration similarly in importance (UK RI = 29.1 vs 29.2, respectively; Brazil RI = 21.3 vs. 21.2, respectively). Variation in the relative importance of other 3L treatment attributes was limited. However, patients from Brazil and Japan continued to value reductions in the risk of neurological events (RI = 20.5 and 15.8, respectively) more than OS and fewer side effects.

## 4 Discussion

We used DCE within geographically diverse samples of treating physicians and patients with R/R FL to evaluate preferences for 2L and 3L treatment attributes. Treatment preferences for physicians and patients were most influenced by PFS. Beyond PFS, patients in the aggregate placed greater emphasis on the route of administration of medications while physicians tended to focus more on five-year OS and safety profiles of agents. Preference for PFS above all other 2L and 3L treatment attributes was consistent among physicians, regardless of practice setting and country. However, variation in treatment preferences relative to country was observed among the patient cohort. Finally, this study further established that the burden of R/R FL is high among patients regarding HRQoL and WPAI. Combined, these results offer key perspectives on how physicians and patients evaluate treatment options in 2L and 3L treatment settings, understanding this information is essential to facilitate shared decision-making in an expanding and complex treatment landscape.

Our results showed physicians and patients preferred treatments with longer PFS time, regardless of the other treatment characteristics assessed. Our findings echo results from previous preference studies in R/R FL treatment. For example, Shafey and colleagues found that treatment preferences among Canadian patients and physicians were most influenced by survival free of relapse time, relative to other treatment characteristics like mode of administration, side effects, and health costs (28). Thomas et al. showed that treatment preferences among US-based patients with R/R FL and treating physicians were most influenced by increases in PFS from 6 months to 36 months (29). We extend this research by corroborating such findings with geographically diverse samples of patients and physicians. Moreover, our DCEs included five-year OS as a treatment attribute, which was also perceived as an influential treatment characteristic, particularly among physicians in the 3L setting. While both PFS and OS suggest survival benefits from treatment, longer periods of stability without disease progression, which are likely to contribute to HRQoL benefits where patients can engage in their daily lives with greater predictability (39), may explain why respondents in our study consistently identified PFS as more influential than OS. Indeed, contemporary research notes that patients prioritize holistic factors when considering their cancer care experiences, including emotional and psychological factors, like hope and opportunities to avoid suffering (40), which may speak to the nuance behind how patients view highly functional living and good health. Combined, our results suggest that PFS is a relevant endpoint for physicians and patients in the context of treatment selection and may be an important topic to discuss while engaging in shared decision-making.

Beyond PFS, physicians and patients differed in their preferences for other treatment attributes. For 2L treatments, patients prioritized safety and favored more convenient administration over five-year OS. Conversely, the influence of safety measures and five-year OS was more balanced among physicians. Five-year OS influenced 3L treatment preferences more for both patients and physicians. Yet, even in the context of 3L treatments, patients continued to place greater importance on administration. This result was largely driven by the inclusion of an administration attribute level in our 3L DCE that was designed to describe ASCT, which uniquely includes a risk of acute graft versus host disease, a condition that can require hospitalization (41). Notably, the attribute level describing ASCT was also least preferred among physicians, but the magnitude of the preference weight was significantly lower than that observed in the patient sample, suggesting that the aversion to ASCT is greater among patients than physicians. As seen in other disease settings, the attractiveness of ASCT likely decreases with increased availability of less toxic and effective. Therefore, we opted for sensitivity analysis with the ASCT level dropped from the administration attribute and found that the influence of administration on patient preferences for 3L treatment decreased, making it more like the influence of OS and risk of neurological events. As expected, administration remained the least influential 3L treatment attribute among physicians.

Compared to physicians, patients showed a stronger preference for 3L treatments with oral tablets over IV infusions. In prior research, Thomas and colleagues also noted that patients found administration/monitoring of R/R FL treatments significantly more important than physicians, and physicians prioritized administration/monitoring below safety outcomes, like CRS and laboratory abnormalities (29). Combined, these results suggest that treatment goals and expectations are likely to differ between patients with R/R FL and their physicians, particularly as they relate to the potential quality of life gains from more convenient modes of administration in later lines of treatment. Therefore, prioritizing effective communication and shared decision-making is essential when selecting appropriate treatment regimens.

Finally, this study builds upon previous literature that shows the burden of R/R FL is high (42–45). In our patient sample, the average EQ-5D index and EQ VAS were lower in each country assessed compared to previously published population norms (46, 47). Approximately one-third of patients experienced work productivity impairment in the form of absenteeism and presenteeism, suggesting that the burden of R/R FL may also have negative societal impacts. Importantly, we noted regional variations in patients’ HRQoL and WPAI measures. Patients from Japan consistently reported comparatively better outcomes across nearly all measures, possibly due to the smaller sample size of Japanese patients and the inclusion of patients who had yet to experience R/R disease due to recruitment challenges. These results underscore the complex interactions between the availability of supportive care services, healthcare systems, and cultural norms that can contribute to geographical variation in patient treatment experiences and outcomes.

### 4.1 Limitations

Participants were recruited via convenience sampling using an online panel that may not reflect the broader population of physicians treating R/R FL and patients with R/R FL. That said, recruitment quotas ensured key segments of the target populations were represented in study sampling. Further, whereas DCE methodology has been shown to provide rigorous and robust insight into treatment decision-making across a host of clinical settings, as with any study utilizing DCE methodology, the treatment profiles and scenarios are hypothetical and therefore may not capture the complexities in decision-making in real-world settings of R/R FL and may be further influenced by experiences and characteristics not evaluating in this study. Finally, our DCE did not include economic factors (e.g., average out-of-pocket costs) in our list of attributes, despite such factors being influential in both patient and physician treatment preferences (28). The decision to exclude such factors was due to the heterogeneity in the role of health systems and health insurance policies across countries assessed. Future studies focused on individual countries would benefit from the inclusion of economic factors in their evaluations of treatment preferences in R/R FL, as such research may further contextualize preferences for more traditional clinical treatment characteristics.

### 4.2 Conclusions

Selecting optimal treatment regimens that meet patients’ individual needs is essential. Our results show that treatments with superior PFS are consistently preferred by patients and physicians. However, physician and patient preferences may diverge when considering other treatment characteristics. For example, physicians may prioritize treatment efficacy in 3L settings while patients may prioritize treatment options that balance efficacy with safety and HRQoL considerations. Our findings support a shared decision-making model in the evolving treatment landscape for R/R FL to ensure individualized treatment planning and optimization. More research is needed to understand the risk/benefit ratio of novel treatment approaches, particularly with the introduction of bispecific antibodies and CAR T treatment.

## Data Availability

The raw data supporting the conclusions of this article will be made available by the authors, without undue reservation.
