# Supplementary material for: Patient and physician treatment preferences in relapsed/refractory follicular lymphoma: a discrete choice experiment in the United States, United Kingdom, France, Germany, Brazil, and Japan
Source: Front Oncol. 2025 Jul 10;15:1589722. doi: 10.3389/fonc.2025.1589722 (PMC12287033; doi:10.3389/fonc.2025.1589722)
Supplement: Supplementary file 1 [file DataSheet1.docx]

Supplementary Material

# Supplementary Methods

The Work Productivity and Activity Impairment – General Health (WPAI-GH) questionnaire (1) was used to assess work-related and non-work-related impairment among patients in our sample.^1^ Following the established scoring protocol (1), absenteeism, presenteeism, and work-productivity impairment scores were derived for patients who were currently employed, whereas overall activity impairment scores were derived for all patients. Scores (0%-100%) are interpreted as the mean percentage of impairment experienced over the past seven days, with higher scores indicating greater impairment.

To evaluate patients’ health-related quality of life (HRQoL) and general health status, the EQ-5D utility index score and EQ Visual Analogue Scale (VAS) were derived from the EQ-5D-5L.^2^ The EQ-5D utility index score is derived from a broader descriptive system that covers domains including mobility, self-care, usual activities, pain/discomfort, and anxiety/depression. Country-specific value sets are applied to generate summary scores that range from 0-1, with a higher score indicating better HRQoL. The EQ VAS is a single item in the EQ-5D-5L system that asks respondents to rate their overall health at that moment, from 0 (worst imaginable health) to 100 (best imaginable health)(31).

The Functional Assessment of Cancer Therapy – General (FACT-G) questionnaire^3^ is a 27-item measure used to evaluate cancer-specific quality of life across four domains: physical, social, emotional, and functional well-being. A total score (range: 0-108) is derived by summing each domain score, with higher scores suggesting better QoL. To capture lymphoma-specific experiences, the FACT-Lymphoma subscale (FACT-LymS) was also administered.^4,5^ This measure includes 15 additional items that complement the FACT-G; these items assess lymphoma-specific symptoms and concerns. FACT-LmyS can be combined with FACT-G total score (0-168) with higher scores indicating better quality of life.

**References**

1. Reilly MC, Zbrozek AS, Dukes EM. The validity and reproducibility of a work productivity and activity impairment instrument. Pharmacoeconomics. 1993;4(5):353-65.
2. Herdman M, Gudex C, Lloyd A, Janssen M, Kind P, Parkin D, et al. Development and preliminary testing of the new five-level version of EQ-5D (EQ-5D-5L). Qual Life Res. 2011;20(10):1727-36.
3. Cella DF, Tulsky DS, Gray G, Sarafian B, Linn E, Bonomi A, et al. The Functional Assessment of Cancer Therapy scale: development and validation of the general measure. J Clin Oncol. 1993;11(3):570-9.
4. Hlubocky FJ, Webster K, Cashy J, Beaumont J, Cella D. The Development and Validation of a Measure of Health-Related Quality of Life for Non-Hodgkin’s Lymphoma: The Functional Assessment of Cancer Therapy—Lymphoma (FACT-Lym). Lymphoma. 2013;2013(1):147176.
5. Hlubocky FJ, Webster K, Beaumont J, Cashy J, Paul D, Abernethy A, et al. A preliminary study of a health-related quality of life assessment of priority symptoms in advanced lymphoma: the National Comprehensive Cancer Network-Functional Assessment of Cancer Therapy - Lymphoma Symptom Index. Leuk Lymphoma. 2013;54(9):1942-6.

# Supplementary Tables

Supplementary Table 1. Mean attribute preference weights in the second-line treatment setting

| **Attributes & Levels** | **Physicians (N=300)** | | | **Patients (N=195)** | | |
| --- | --- | --- | --- | --- | --- | --- |
|  | **Mean** | **(95% CI)** | **Δ** | **Mean** | **(95% CI)** | **Δ** |
| PFS |  |  |  |  |  |  |
| 1 year, 8 months | -1.84 | (-1.99, -1.69) | 4.32 | -2.12 | (-2.29, -1.95) | 4.49 |
| 2 years, 2 months | -0.63 | (-0.69, -0.58) |  | -0.25 | (-0.34, -0.15) |  |
| 3 years, 9 months | 2.48 | (2.30, 2.65) |  | 2.37 | (2.16, 2.58) |  |
| 5-year OS |  |  |  |  |  |  |
| 55% | -1.27 | (-1.40, -1.14) | 2.46 | -0.55 | (-0.65, -0.46) | 1.31 |
| 65% | 0.08 | (0.02, 0.15) |  | -0.21 | (-0.27, -0.15) |  |
| 75% | 1.19 | (1.09, 1.29) |  | 0.76 | (0.66, 0.86) |  |
| Serious adverse events |  |  |  |  |  |  |
| 25% | 0.48 | (0.42, 0.54) | 0.95 | 0.54 | (0.46, 0.61) | 1.02 |
| 35% | -0.02 | (-0.07, 0.03) |  | -0.05 | (-0.11, 0.00) |  |
| 45% | -0.46 | (-0.52, -0.40) |  | -0.48 | (-0.55, -0.42) |  |
| CRS events (any grade) |  |  |  |  |  |  |
| 0% | 1.19 | (1.11, 1.27) | 2.44 | 1.12 | (1.00, 1.23) | 2.07 |
| 40% | 0.06 | (0.01, 0.11) |  | -0.16 | (-0.24, -0.09) |  |
| 78% | -1.25 | (-1.32, -1.18) |  | -0.95 | (-1.10, -0.80) |  |
| Neurological events (any grade) |  |  |  |  |  |  |
| 0% | 1.45 | (1.34, 1.57) | 2.79 | 1.55 | (1.35, 1.75) | 3.13 |
| 30% | -0.11 | (-0.17, -0.06) |  | 0.03 | (-0.02, 0.08) |  |
| 56% | -1.34 | (-1.44, -1.24) |  | -1.58 | (-1.76, -1.40) |  |
| Fatigue (any grade) |  |  |  |  |  |  |
| 0% | 0.72 | (0.65, 0.79) | 1.37 | 0.62 | (0.49, 0.76) | 1.27 |
| 25% | -0.07 | (-0.12, -0.03) |  | 0.02 | (-0.05, 0.10) |  |
| 45% | -0.65 | (-0.72, -0.57) |  | -0.65 | (-0.77, -0.53) |  |
| Administration |  |  |  |  |  |  |
| Blood is collected from the patient. 2-3 weeks later, the patient is admitted to the hospital to receive 3 days of IV infusion, followed by another IV infusion. Patients remain in hospital for an additional week. After 4 weeks patients return to normal functionin.^1^ | -0.05 | (-0.10, -0.01) | 0.92 | 0.16 | (0.02, 0.30) | 2.14 |
| Blood is collected from the patient. The patient receives IV infusion and remains in the hospital for 2-3 weeks. After 3-6 months patients return to normal functionin.^2^ | -0.44 | (-0.51, -0.37) |  | -0.24 | (-0.35, -0.13) |  |
| The patient receives IV infusion during an outpatient visit 2 days every 4 weeks for 6 months^3^ | 0.48 | (0.41, 0.55) |  | 1.11 | (0.95, 1.27) |  |
| The patient receives IV infusion during an outpatient visit every week for 4 weeks, followed by IV infusion 2 days every 4 weeks for 6 months, followed by one IV infusion every 2 months for 2 years^4^ | 0.01 | (-0.04, 0.07) |  | -1.03 | (-1.21, -0.86) |  |
| Abbreviations: 2L= second line of therapy; CI = Confidence Interval; CRS= Cytokine Release Syndrome; IV= intravenous; OS= overall survival; PFS=progression free survival; SE = Standard Error  Note: Preference weights should not be interpreted by themselves. Instead, the magnitude of change within one attribute (Δ) should be compared to change within another attribute. All preference weights of levels within an attribute sum to 0.  ^1^Characterizes the mode of administration associated with CAR-T therapy  ^2^Characterizes the mode of administration associated with Autologous Cell Transplant  ^3^Characterizes the mode of administration associated with rituximab, bendamustine  ^4^Charaterizes the mode of administration associated with obinutuzumab, bendamustine | | | | | |  |
|  | | | | | |  |
|  | | | | | |  |

Supplementary Table 2. Mean attribute preference weights in the third-line treatment setting

| **Attributes & Levels** | **Physicians (N=300)** | | | **Patients (N=195)** | | |
| --- | --- | --- | --- | --- | --- | --- |
|  | **Mean** | **(95% CI)** | **Δ** | **Mean** | **(95% CI)** | **Δ** |
| PFS |  |  |  |  |  |  |
| 10 months | -3.50 | (-3.82, -3.18) | 6.64 | -3.34 | (-3.68, -3.01) | 6.11 |
| 2 years | 0.36 | (0.29, 0.42) |  | 0.57 | (0.47, 0.67) |  |
| 3 years, 3 months | 3.14 | (2.83, 3.45) |  | 2.77 | (2.51, 3.04) |  |
| 5-year OS |  |  |  |  |  |  |
| 43% | -2.38 | (-2.62, -2.15) | 4.29 | -1.07 | (-1.26, -0.89) | 2.28 |
| 65% | 0.48 | (0.42, 0.53) |  | -0.14 | (-0.22, -0.05) |  |
| 74% | 1.91 | (1.71, 2.10) |  | 1.21 | (1.08, 1.34) |  |
| Serious adverse events |  |  |  |  |  |  |
| 27% | 0.43 | (0.36, 0.51) | 0.80 | 0.89 | (0.78, 1.01) | 1.66 |
| 45% | -0.06 | (-0.11, -0.01) |  | -0.13 | (-0.18, -0.08) |  |
| 58% | -0.37 | (-0.42, -0.32) |  | -0.77 | (-0.86, -0.67) |  |
| CRS events (any grade) |  |  |  |  |  |  |
| 0% | 0.51 | (0.42, 0.59) | 1.14 | 0.82 | (0.72, 0.91) | 1.95 |
| 40% | 0.12 | (0.06, 0.18) |  | 0.31 | (0.25, 0.37) |  |
| 78% | -0.63 | (-0.71, -0.55) |  | -1.13 | (-1.23, -1.03) |  |
| Neurological events (any grade) |  |  |  |  |  |  |
| 0% | 1.31 | (1.20, 1.42) | 2.62 | 1.17 | (1.03, 1.31) | 2.22 |
| 30% | -0.01 | (-0.05, 0.03) |  | -0.12 | (-0.22, -0.02) |  |
| 56% | -1.31 | (-1.40, -1.21) |  | -1.05 | (-1.23, -0.87) |  |
| Fatigue (any grade) |  |  |  |  |  |  |
| 0% | 0.54 | (0.47, 0.62) | 1.28 | 0.61 | (0.45, 0.76) | 1.63 |
| 15% | 0.22 | (0.15, 0.29) |  | 0.48 | (0.39, 0.57) |  |
| 30% | -0.03 | (-0.10, 0.04) |  | -0.07 | (-0.17, 0.04) |  |
| 45% | -0.74 | (-0.85, -0.62) |  | -1.02 | (-1.19, -0.86) |  |
| Administration |  |  |  |  |  |  |
| Blood is collected from the patient. 2-3 weeks later, the patient is admitted to the hospital to receive 3 days of IV infusion, followed by another IV infusion. Patients remain in hospital for an additional week. After 4 weeks patients return to normal functioning^1^ | 0.10 | (0.01, 0.19) | 1.29 | 0.11 | (-0.05, 0.27) | 5.73 |
| Blood is collected from the patient. The patient receives IV infusion and remains in the hospital for 2-3 weeks. After 3-6 months patients return to normal functioning^2^ | -0.12 | (-0.22, -0.02) |  | -0.10 | (-0.19, -0.01) |  |
| Blood is collected from the patient. The patient receives IV infusion and remains in the hospital for 2-4 weeks. After 6-12 months patients return to normal functioning. Patients are at risk for developing a serious condition that most often occurs within the first 100 days but can occur years after the procedure and often requires hospitalization^3^ | -0.78 | (-0.85, -0.72) |  | -3.62 | (-3.92, -3.33) |  |
| Tablet is taken by mouth twice a day for 2 years^4^ | 0.51 | (0.41, 0.61) |  | 2.11 | (1.93, 2.29) |  |
| The patient receives IV infusion during an outpatient visit every week for 3 weeks, followed by IV infusion every 3 weeks for one year^5^ | 0.15 | (0.09, 0.21) |  | 0.11 | (-0.02, 0.25) |  |
| The patient receives IV infusion during an outpatient visit 2 days every 4 weeks for 6 months^6^ | 0.14 | (0.06, 0.23) |  | 1.38 | (1.21, 1.56) |  |
| Abbreviations: 3L = third line of therapy; CI = Confidence Interval; CRS= Cytokine Release Syndrome; IV= intravenous; OS= overall survival; PFS=progression free survival; SE = Standard Error  Preference weights should not be interpreted by themselves. Instead, the magnitude of change within one attribute (Δ) should be compared to change within another attribute. All preference weights of levels within an attribute sum to 0.  ^1^Characterizes the mode of administration associated with CAR-T therapy  ^2^Characterizes the mode of administration associated with Autologous Cell Transplant  ^3^Charterizes the mode of administration associated with Allogeneic Stem Cell Transplant  ^4^Characterizes the mode of administration associated with Tazemetostat / Idelalisib / Duvelisib  ^5^Characterizes the mode of administration associated with mosunetuzumab-axgb  ^6^Charaterizes the mode of administration associated with obinutuzumab, bendamustine | | | | | | |

Supplementary Table 3. Mean preference weights, 95% CIs, and absolute difference between most and least preferred attributes from the third-line discrete choice experiment with the allogeneic stem cell transplant attribute level removed

| **Attributes & Levels** | **Physicians (N=300)** | | | **Patients (N=195)** | | |
| --- | --- | --- | --- | --- | --- | --- |
|  | **Mean** | **(95% CI)** | **Δ** | **Mean** | **(95% CI)** | **Δ** |
| PFS |  |  |  |  |  |  |
| 10 months | -3.5 | (-3.82, -3.18) | 6.64 | -3.34 | (-3.68, -3.01) | 6.11 |
| 2 years | 0.36 | (0.29, 0.42) |  | 0.57 | (0.47, 0.67) |  |
| 3 years, 3 months | 3.14 | (2.83, 3.45) |  | 2.77 | (2.51, 3.04) |  |
| 5-year OS |  |  |  |  |  |  |
| 43% | -2.38 | (-2.62, -2.15) | 4.29 | -1.07 | (-1.26, -0.89) | 2.28 |
| 65% | 0.48 | (0.42, 0.53) |  | -0.14 | (-0.22, -0.05) |  |
| 74% | 1.91 | (1.71, 2.10) |  | 1.21 | (1.08, 1.34) |  |
| Serious adverse events |  |  |  |  |  |  |
| 27% | 0.43 | (0.36, 0.51) | 0.80 | 0.89 | (0.78, 1.01) | 1.66 |
| 45% | -0.06 | (-0.11, -0.01) |  | -0.13 | (-0.18, -0.08) |  |
| 58% | -0.37 | (-0.42, -0.32) |  | -0.77 | (-0.86, -0.67) |  |
| CRS events (any grade) |  |  |  |  |  |  |
| 0% | 0.51 | (0.42, 0.59) | 1.14 | 0.82 | (0.72, 0.91) | 1.95 |
| 45% | 0.12 | (0.06, 0.18) |  | 0.31 | (0.25, 0.37) |  |
| 78% | -0.63 | (-0.71, -0.55) |  | -1.13 | (-1.23, -1.03) |  |
| Neurological events (any grade) |  |  |  |  |  |  |
| 0% | 1.31 | (1.20, 1.42) | 2.62 | 1.17 | (1.03, 1.31) | 2.22 |
| 30% | -0.01 | (-0.05, 0.03) |  | -0.12 | (-0.22, -0.02) |  |
| 56% | -1.31 | (-1.40, -1.21) |  | -1.05 | (-1.23, -0.87) |  |
| Fatigue (any grade) |  |  |  |  |  |  |
| 0% | 0.54 | (0.47, 0.62) | 1.28 | 0.61 | (0.45, 0.76) | 1.63 |
| 15% | 0.22 | (0.15, 0.29) |  | 0.48 | (0.39, 0.57) |  |
| 30% | -0.03 | (-0.10, 0.04) |  | -0.07 | (-0.17, 0.04) |  |
| 45% | -0.74 | (-0.85, -0.62) |  | -1.02 | (-1.19, -0.86) |  |
| Administration |  |  |  |  |  |  |
| Blood is collected from the patient. 2-3 weeks later, the patient is admitted to the hospital to receive 3 days of IV infusion, followed by another IV infusion. Patients remain in hospital for an additional week. After 4 weeks patients return to normal functioning^1^ | -0.06 | (-0.15, 0.03) | 0.62 | -0.61 | (-0.77, -0.45) | 2.21 |
| Blood is collected from the patient. The patient receives IV infusion and remains in the hospital for 2-3 weeks. After 3-6 months patients return to normal functioning^2^ | -0.27 | (-0.37, -0.18) |  | -0.82 | (-0.91, -0.73) |  |
| Tablet is taken by mouth twice a day for 2 years^3^ | 0.35 | (0.26, 0.44) |  | 1.39 | (1.21, 1.57) |  |
| The patient receives IV infusion during an outpatient visit every week for 3 weeks, followed by IV infusion every 3 weeks for one year^4^ | -0.01 | (-0.07, 0.06) |  | -0.61 | (-0.75, -0.48) |  |
| The patient receives IV infusion during an outpatient visit 2 days every 4 weeks for 6 months^5^ | -0.01 | (-0.09, 0.07) |  | 0.66 | (0.48, 0.83) |  |
| Abbreviations: 3L = third line of therapy; CI = Confidence Interval; CRS= Cytokine Release Syndrome; IV= intravenous; OS= overall survival; PFS=progression free survival; SE = Standard Error  Preference weights should not be interpreted by themselves. Instead, the magnitude of change within one attribute should be compared to change within another attribute. All preference weights of levels within an attribute sum to 0.  ^1^Characterizes the mode of administration associated with CAR T therapy  ^2^Characterizes the mode of administration associated with Autologous Cell Transplant  ^3^Characterizes the mode of administration associated with Tazemetostat / Idelalisib / Duvelisib  ^4^Characterizes the mode of administration associated with mosunetuzumab-axgb  ^5^Charaterizes the mode of administration associated with obinutuzumab, bendamustine | | | | | | |

# Supplementary Figures

**Supplementary Figure 1.** Mean preference weights for attributes associated with third-line treatment for relapsed/refractory follicular lymphoma with the allogeneic stem cell transplant attribute level removed from administration

Abbreviations: CRS= Cytokine Release Syndrome; IV= intravenous; OS= overall survival; PFS=progression free survival

Note: Preference weights should not be interpreted by themselves. Instead, the magnitude of change within one attribute should be compared to change within another attribute. All preference weights of levels within an attribute sum to 0.

*Blood is collected from the patient. 2-3 weeks later, the patient is admitted to the hospital to receive 3 days of IV infusion, followed by another IV infusion. Patients remain in the hospital for an additional week. After 4 weeks patients return to normal functioning

**Blood is collected from the patient. The patient receives IV infusion and remains in the hospital for 2-3 weeks. After 3-6 months patients return to normal functioning

***Tablet is taken by mouth twice a day for 2 years

^ⱡ^The patient receives IV infusion during an outpatient visit every week for 3 weeks, followed by IV infusion every 3 weeks for one year.

^ⱡⱡ^The patient receives IV infusion during an outpatient visit 2 days every 4 weeks for 6 months.
